# Prenatal ultrasonographic features and follow-up outcomes of 19 cases of congenital intrahepatic portosystemic venous shunts diagnosed during the foetal period

**DOI:** 10.1186/s13244-022-01310-8

**Published:** 2022-10-20

**Authors:** Linlin Zhu, Haifang Wu, Xiang Cong, Shizhen Li, Qi Li, Xiangyi Dong, Guowei Tao

**Affiliations:** Department of Ultrasound, Qilu Hospital of Shandong University, 107 Wenhua West Road, Jinan, 250012 China

**Keywords:** Ultrasonography, Congenital intrahepatic portosystemic venous shunts, Prenatal diagnosis, Prognosis

## Abstract

**Background:**

To investigate the prenatal ultrasonographic features and case characteristics of the congenital intrahepatic portosystemic venous shunt (IHPSS) diagnosed during the foetal period and analyse its prognosis.

**Methods:**

We conducted a retrospective cohort study of patients diagnosed with IHPSS between 2016 and 2021. IHPSS was defined as an abnormal connection between the foetal intrahepatic portal and the hepatic veins.

**Results:**

In this study, 19 foetuses were identified, including 12 cases of single shunt and 7 cases of multiple shunts, with a gestational age of 33.8 ± 4.5 (range 25–40) weeks at diagnosis. In the single-shunt group, the origin position of the shunts was all from the left branch of the portal vein (LPV), whereas in the multiple-shunt group, the origin position of the shunts was from the LPV in six cases. Common concomitant intrauterine abnormalities of IHPSS include foetal growth restriction (47.4%) and foetal cardiac enlargement (21.1%). The postnatal manifestations of IHPSS include biochemical abnormalities (increased gamma-glutamyl transferase and bilirubin levels), neonatal hypoglycaemia, neonatal hyperammonaemia, pulmonary hypertension, multiple intrahepatic hyperechoic nodules, and cutaneous haemangiomas. Spontaneous closure of shunts occurred in ten cases, and the mean time to shunt closure was 8.1 months (1–28 months).

**Conclusions:**

Most IHPSS found during the foetal period is located in the left branch of the portal vein, and the gestational age at diagnosis is usually in the late second or third trimester. Spontaneous closure of shunts can occur in most live births, and the prognosis is good.

## Key points

- Most IHPSSs during the foetal period are located in the left lobe.
- The most common intrauterine concomitant abnormality in IHPSS was foetal growth restriction.
- Spontaneous closure of shunts can occur in most live births.

## Background

Intrahepatic portosystemic shunt (IHPSS), characterised by abnormal connections between the intrahepatic portal vein branches and the hepatic vein, is a rare vascular malformation. Most previous studies of congenital portosystemic shunts have focused on children and adults [1, 2]. However, these studies have limited clinical relevance for foetal IHPSS due to the foetus’s unique anatomical and circulatory characteristics.

With the development of prenatal ultrasonography, some articles have also reported IHPSS in the foetus [3, 4]; however, little is known about its intrauterine characteristics and prognosis. This study aimed to review our experience in diagnosing IHPSS during the foetal period, analyse their clinical features and prognosis, and provide assistance for perinatal management and prenatal counselling.

## Methods

All foetal IHPSS cases diagnosed at our department between April 2016 and September 2021 were reviewed. The inclusion criterion was an intrauterine diagnosis of the IHPSS, which is an abnormal connection between the foetal intrahepatic portal vein and the hepatic vein. A part of this series was mentioned in our previous study [5].

Ultrasound examinations were performed using PHILIPS iU22 or Philips EPIQ7 colour Doppler ultrasound instruments equipped with a C5-1 broadband curved array transducer and an eL18-4 ultra-broadband linear array transducer.

The foetuses underwent detailed ultrasonography, including echocardiography. According to the method proposed by Yagel [6], the foetal umbilical–portal system was evaluated. Additionally, the foetal umbilical vein, hepatic vein, intrahepatic portal vein and branches, main portal vein, and venous duct were examined and recorded, and the location and shape of the shunt were documented. The gestational age (GA) at the screening time was based on the crown–rump length (CRL) measured in the first trimester.

Follow-up was completed by two doctors, which included delivery mode, neonatal weight, postnatal abdominal ultrasound examination, CT examination, biochemical examination, and chromosome examination.

Statistical analysis was performed using SPSS 26 (IBM SPSS Statistics, New York, NY, USA) and Microsoft Excel 2019 (Microsoft Corp., Richmond, CA, USA) software. Mean and standard deviation values were used for descriptive statistics. For nonparametric variables (cases of foetal growth restriction in the single- and multiple-shunt groups), we used Fisher’s exact test, given the few cases, and a *p* < 0.05 was considered statistically significant.

## Results

A total of 19 foetuses were diagnosed with IHPSS during the study period. Patient characteristics are presented in Table 1. The maternal age was 29.9 ± 5.3 (range 22–42) years, and the gestation age at diagnosis was 33.8 ± 4.5 (range 25–40) weeks. Excluding one case of twin pregnancy, the rest were singleton pregnancies.

**Table 1:** Patient characteristics of 19 foetuses diagnosed with IHPSS during the study period

Case	Maternal age (year)	GA (week)	Single or multiple shunts	Position of shunts	Cystic expansion at the shunt	Regular or irregular multiple shunts	FGR	Associated anomalies	Gender	Outcome	Delivery mode	Delivery gestational age (week)	Birth weight (g)	Surgical treatment	Spontaneous closure of shunt	Time to shunt closure (month)
1	26	36	SS	LPVi-LHV	Yes	–	Yes	None	Female	LB	CS	39	2400	No	YES	16
2	28	28	SS	LPVi-MHV	Yes	–	No	None	Male	LB	VD	38	Unknown	Yes	–	–
3	38	33	SS	LPVi-LHV	No	–	Yes	None	Female	LB	CS	35	1400	No	Yes	5
4	29	32	SS	LPVi-LHV	No	–	Yes	None	Male	LB	CS	37	1710	No	Yes	28
5	26	34	SS	LPVi-LHV	No	–	Yes	None	Unknown	TOP	–	–	–	–	–	–
6	35	28	SS	LPVi-LHV	No	–	No	None	Female	LB	CS	40	3240	No	Yes	8
7	29	38	SS	LPVi-LHV	No	–	Yes	CE;EIF	Unknown	TOP	–	–	–	–	–	–
8	42	36	SS	LPVm-RHV	Yes	–	Yes	None	Male	LB	CS	39	Unknown	No	Yes	1
9	32	26	SS	LPVi-LHV	No	–	No	None	Female	LB	CS	40	3400	No	Yes	14
10	38	39	SS	LPVs-LHV	Yes	–	No	None	Male	LB	VD	39	3530	Unknown	Unknown	–
11	23	31	SS	LPVi-LHV	No	–	Yes	DI	Unknown	LB	CS	Unknown	Unknown	Unknown	Unknown	–
12	25	32	SS	LPVm-MHV	No	–	Yes	CE	Unknown	TOP	–	–	–	–	–	–
13	32	37	MS	LPVi-LHV LPVs-LHV LPVm-MHV	No	Regular	No	CE;SM	Male	LB	CS	38	3000	No	Yes	3
14	30	25	MS	LPVi-LHV LPVs-LHV LPVm-MHV	No	Irregular	No	None	Unknown	TOP	–	–	–	–	–	–
15	25	35	MS	LPVi-LHV LPVs-LHV	Yes	Irregular	Yes	SM	Female	TOP	–	–	–	–	–	–
16	31	37	MS	LPVi-LHV LPVs-LHV	No	Regular	No	HSM	Male	LB	CS	38	Unknown	No	Yes	3
17	29	38	MS	LPVi-LHV LPVm-MHV	Yes	Irregular	No	None	Male	LB	CS	39	2600	No	Yes	1
18	29	37	MS	LPVm-MHV	No	Regular	No	None	Female	LB	Unknown	39	Unknown	No	Yes	2
19	22	40	MS	PS-IVC LPVs-LHV LPVm-MHV ARPV-RHV ARPV-MHV PRPV-RHV	No	Regular	NO	CE	Female	LB	VD	40	4500	Yes	–	–

*GA* gestational age at diagnosis, *SS* single shunt, *MS* multiple shunts, *LPVi* inferior left portal vein, *LPVs* superior left portal vein, *LPVm* medial left portal vein, *ARPV* anterior right portal vein, *PRPV* posterior right portal vein, *LHV* left hepatic vein, *MHV* middle hepatic vein, *RHV* right hepatic vein, *PS* portal sinus, *IVC* inferior vena cava, *FGR* foetal growth restriction, *CE* cardiac enlargement, *SM* splenomegaly, *EIF* Echogenic intracardiac focus, *HSM* hepatosplenomegaly, *DI* duodenal ileus, *LB* live birth, *TOP* termination of pregnancy, *CS* caesarean section, *VD* vaginal delivery

**Fig. 1:**
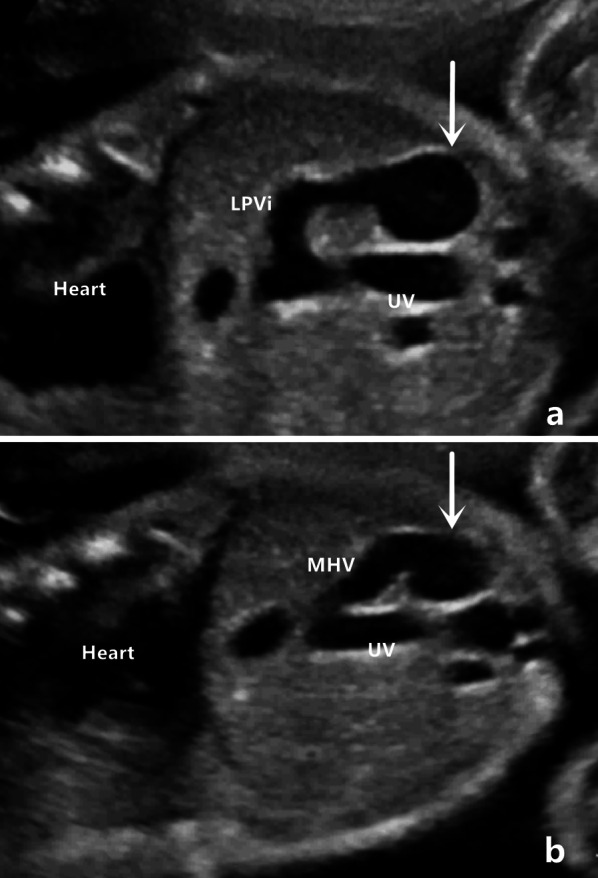
Cystic expansion at the shunt. This figure shows a shunt between the inferior left portal vein (LPVi) and middle hepatic vein (MHV). The arrow points to cystic expansion at the shunt. UV: umbilical vein

Based on the number of shunts between the hepatic and intrahepatic portal veins, the cases were divided into single-shunt (12 cases) and multiple-shunt (7 cases) groups. Ultrasound images of shunts vary, including cystic expansion at the shunt, and multiple shunts can be regular or irregular (Fig. 1).

In the single-shunt group, the origin position of the shunts was all from the left branch of the portal vein (LPV), including nine cases of the inferior LPV (LPVi), two cases of the medial LPV (LPVm), and one case of the superior LPV (LPVs).

**Fig. 2:**
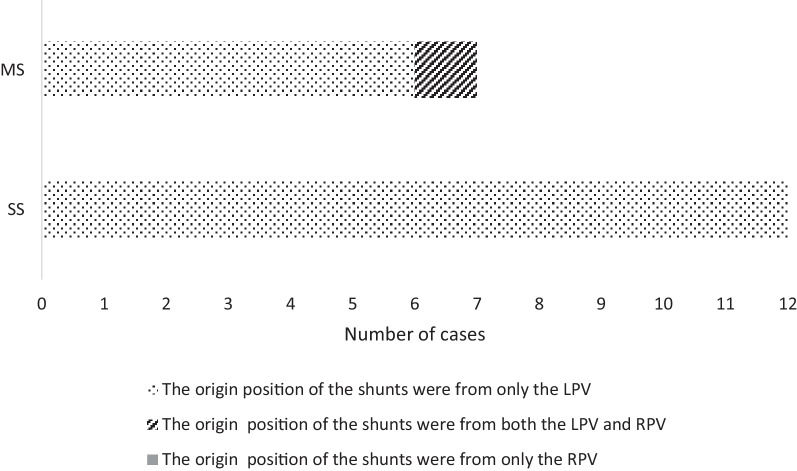
In the single-shunt group, the origin position of the shunts was all from the LPV. In the multiple-shunt group, the origin position of the shunts was from the LPV in six cases, and both the LPV and RPV in one case. There was no case that the origin position of the shunts was from only the RPV. SS: single shunt; MS: multiple shunts; LPV: left portal vein; RPV: right portal vein

In the multiple-shunt group, the origin position of the shunts was from the LPV in six cases, and the origin position of the shunts included both the left branch of the portal vein and the right branch of the portal vein in one case. This patient also had an extrahepatic portosystemic shunt (between the portal sinus and inferior vena cava) (Fig. 2).

Several associated anomalies were detected; the most common intrauterine concomitant abnormality was foetal growth restriction (FGR) (nine cases), followed by cardiac enlargement (four cases). Accompanying abdominal organ abnormalities that we found prenatally included two cases of splenomegaly, one case of hepatosplenomegaly, and one case of duodenal ileus (Table 1).

Postnatal complications included four cases of neonatal hypoglycaemia, nine cases of elevated gamma-glutamyl transferase (GGT), nine cases of neonatal bilirubin elevation, and six cases of neonatal hyperammonaemia. On echocardiography, two neonates were diagnosed with pulmonary hypertension, and both resolved during follow-up. Two neonates were found to have multiple intrahepatic hyperechoic nodules during postnatal liver ultrasonography, which spontaneously subsided during the follow-up. These two cases were in the multiple-shunt group, one of which was intrahepatic with extrahepatic portosystemic shunt, while the neonate also developed multiple cutaneous haemangiomas that resolved spontaneously.

**Fig. 3:**
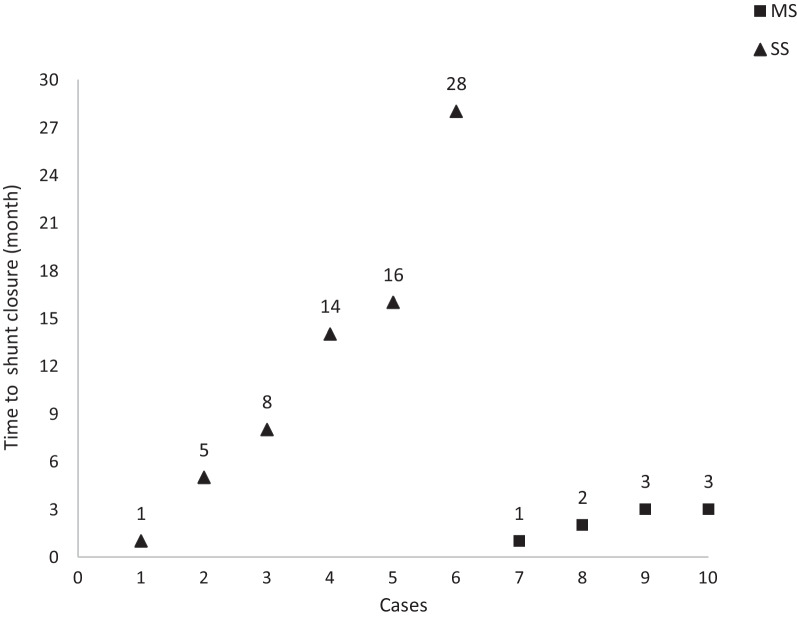
Spontaneous closure of shunts occurred in ten conservatively treated children, including six cases in the single-shunt group and four cases in the multiple-shunt group. SS: single shunt; MS: multiple shunts

Two patients were lost to follow-up. Among the remaining 12 cases (7 cases in the single-shunt group and 5 cases in the multiple-shunt group), 10 patients received conservative treatment and 2 patients received surgical treatment. Spontaneous closure of shunts occurred in all ten conservatively treated children, including six cases in the single-shunt group and four cases in the multiple-shunt group. The mean time to shunt closure was 8.1 months (1–28 months) (Fig. 3). Two children received surgical treatment, one of whom was in the single-shunt group. Laparoscopic portal vein–hepatic vein fistula ligation was performed 12 days after birth, and the shunt was closed after the operation. Another case involved intrahepatic and extrahepatic shunts. Right portal vein ligation was performed at 4 months old; however, there remained a partial intrahepatic shunt after the operation, which was completely closed at 19 months old.

In two cases of labour induction, copy number variations (CNVs) were found during the examination of labour induction specimens. These two cases were in the single-shunt group: one case showed seq[hg19] 7q11.23 (72.74 Mb-74.16 Mb) × 1, and the other case showed 46,XN,dup (Xp11.23).seq[GRCh37/hg19](47,854,066–47,983,254) × 3. Karyotype analysis of the peripheral blood of one live birth infant (multiple-shunt group) showed 46,XY,inv(9),p12q13.

## Discussion

Since Abernethy first discovered the congenital portosystemic shunt (CPSS) in 1793 [7], an increasing number of studies have been conducted on this disease [8–11]. According to the location of the shunt between the portal and systemic veins, the CPSS can be divided into intrahepatic and extrahepatic types and various subtypes. The previous description of foetal CPSS also used the adult classification until Achiron proposed the foetal umbilical–portal–systemic shunt (UPSVS) classification [12], including type I umbilical–systemic shunt, type II ductus venosus–systemic shunt, and type III portal–systemic shunt. Type III is further divided into two subtypes: type IIIa, intrahepatic portal–systemic shunt, and type IIIb, extrahepatic portal–systemic shunt. This classification is currently the only foetal type of CPSS that can better reflect the unique blood circulation characteristics of the foetal period and is more suitable for the diagnosis of foetal CPSS. However, prenatal ultrasound manifestations of type IIIa are diverse, and no further elaboration has been made regarding the characteristics of this type. It must be emphasised that prenatal classification is not the same as postnatal classification. Furthermore, for optimal treatment and management after birth, various imaging modalities, including CT angiography and Doppler ultrasound, should be considered to precisely define the involved vessels, type of shunt, and presence of aneurysm/dilation [13].

The direct manifestation of the congenital IHPSS is an abnormal connection between the hepatic and intrahepatic portal veins. Due to the limitations of imaging conditions, prenatal classification of IHPSS based on the Park classification is unreliable [10]. However, it is not difficult to distinguish single or multiple shunts by prenatal ultrasound. The abnormal connection was often close to the liver capsule (78.9%, 15/19), indicating that the shunts were mostly located in the peripheral branches of the hepatic and intrahepatic portal veins. The morphology of the abnormal connection was diverse: 33.3% (4/12) of the single-shunt group and 28.6% (2/7) of the multiple-shunt group showed cystic dilatation at the shunt. In the multiple-shunt group, 57.1% had irregular shunts and 42.9% had regular shunts.

In our experience, most IHPSS cases diagnosed in the foetal period, whether single or multiple shunts, mostly originate in the left portal branch on the portal side. In the single-shunt group, 100% (12/12) originated from the left portal branch, of which 75% (9/12) were in the LPVi, 16.7% (2/12) in the LPVm, and 8.3% (1/12) in the LPVs (Fig. 2). In the multiple-shunt group, excluding one patient with both intrahepatic and extrahepatic shunts, this patient had abnormal communications between the left portal vein and the hepatic vein and between the right portal vein and the hepatic vein, the remaining shunts all originated from the left portal vein. On the hepatic vein side, the most common location of IHPSS is the left hepatic vein (LHV). In the single-shunt group, 75% of the shunts were located in the LHV, 16.7% in the middle hepatic vein (MHV), and 8.3% in the right hepatic vein (RHV). In the multiple-shunt group, 85.7% (6/7) of the cases included the LHV shunts, 71.4% (5/7) included MHV shunts, and 14.3% (1/7) included RHV shunts.

We found that the left lobe of the IHPSS diagnosed in the foetal period was more common than the right lobe, whether on the portal or hepatic venous side. This conclusion is consistent with that reported by Kivilevitch et al. [4]. In their cases, except for one foetus in which the left portal vein could not be identified, the starting position of the shunt in all other cases was located in the left portal vein. Cytter-Kuint et al. studied IHPSS in 15 children, of which 14 shunts were located between the LPV and hepatic vein, and only one case was located between the RPV and RHV [14]. However, none of these studies have discussed this feature further.

The hepatic primordium emerges in the fourth week of gestation and is in contact with the vitelline and umbilical venous systems. Eventually, liver blood vessels are formed via angiogenesis, vasculogenesis, and vascular differentiation [15]. The segments of the vitelline veins located between the subdiaphragmatic and subhepatic anastomoses are interrupted by the developing hepatic trabeculae and resolve into a plexus of small vessels connected to the hepatic sinusoids. The hepatic afferent veins (venæ advehentes) convey blood from the subhepatic anastomosis to the sinusoidal plexus, while the hepatic efferent veins (venæ revehentes) drain blood from the sinusoidal plexus to the subdiaphragmatic anastomosis [16–18]. The foetal umbilical vein completely supplies the left portal vein of the foetal liver, whereas the right portal vein of the foetus is supplied by both the umbilical vein and the main portal vein. The blood flow in the left portal vein of the foetal liver was greater than that in the right portal vein [19, 20]. IHPSS is a vascular malformation caused by incomplete vascular remodelling of the vitelline vein. We speculate that in the process of gradual remodelling of the small vascular plexus connected to the sinus plexus into the peripheral branches of the portal vein and hepatic vein, the peripheral branch of the LPV has greater blood flow because the LPV connects to the umbilical vein, making it easier to “maintain” the abnormal connection with the peripheral branch of the hepatic vein. That is, increased blood flow facilitates the formation of an IHPSS. This may be the reason the IHPSS we observed during the foetal period was more common in the left branch of the portal vein. In the single-shunt group, the closer the portal vein branch was to the umbilical vein, the more shunts were observed (LPVi > LPVm > LPVs). The spontaneous closure of IHPSS diagnosed prenatally after birth may be related to the closure of the umbilical vein and the decrease in portal venous blood flow after birth, which, on the other hand, indicates that low blood flow is not conducive to the “maintenance” of the abnormal shunt.

**Fig. 4:**
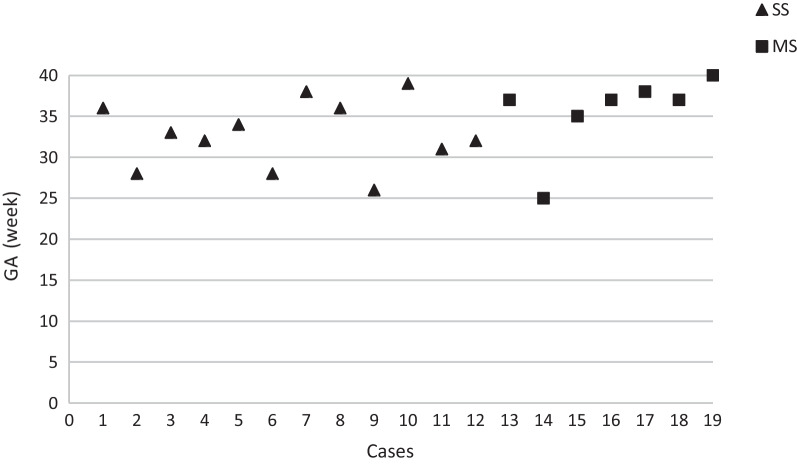
The gestational age at diagnosis of nineteen cases. Note that all cases were diagnosed in the late second and third trimesters. GA: gestational age at diagnosis; SS: single shunt; MS: multiple shunts

The mean gestational age at diagnosis in our cases was 33.8 ± 4.5 weeks (25–40 weeks), which is consistent with the conclusions of other researchers [4, 21] that IHPSS is mostly diagnosed in the late second and third trimesters (Fig. 4). The mean gestational age at diagnosis was 35.6 ± 4.9 weeks in the multiple-shunt group and 32.8 ± 4.1 weeks in the single-shunt group. Kivilevitch Z pointed out that the reasons leading to late diagnosis include the relatively small amount and low velocity of the blood flowing in these venous systems in the first trimester, lack of disease awareness by examiners, and acquired late formation of the shunts [4]. They believe that the two cavernous shunts in their cases were caused by vascular thrombotic events; thus, the shunts were discovered in the third trimester. In our case, no similar evidence of acquired shunts (e.g. intrahepatic calcifications that appeared until mid-pregnancy) was observed.

The most common intrauterine concomitant abnormality in IHPSS was FGR, with an incidence of 47.4%. No significant difference was observed in the incidence of FGR between the single and multiple groups (Table 2). In foetal sheep, experimental reduction of umbilical vein hepatic perfusion results in the decreased foetal liver synthesis of insulin-like growth factors, impairing foetal growth [22]. Therefore, abnormal shunting of the left portal vein and the hepatic vein leads to a decrease in blood perfusion in the liver, which may cause FGR in IHPSS.

**Table 2:** Cases of FGR in the single- and multiple-shunt groups

Type of shunts	FGR	No FGR	Total
SS	8	4	12
MS	1	6	7
Total	9	10	19

No significant difference was observed in the incidence of FGR between the single- and multiple-shunt groups. The p value of Fisher’s exact test (2-sided) was 0.057

*FGR* foetal growth restriction, *SS* single shunt, *MS* multiple shunts

Another common intrauterine abnormality is cardiac enlargement, with an incidence rate of 21.1%. Abnormal shunts lead to increased cardiac preload and, thus, enlargement of the heart, which suggests that attention should be given to evaluating cardiac function in patients with IHPSS. Some researchers have proposed the potential benefit of f-TAPSE (tricuspid annular plane contraction drift) for the longitudinal monitoring of growth-restricted foetuses diagnosed concomitantly with cardiomegaly and CPSS [23].

In our case, there was no significant difference in the incidence between boys and girls, and in cases of known sex, the male-to-female ratio was 1:1 for both the single and multiple groups. There were 14 live births (73.7%, 14/19), with 75% in the single-shunt group and 71.4% in the multiple-shunt group. Thirteen cases were followed up for the mode of delivery; caesarean section accounted for the vast majority (76.9%, 10/13), and the average gestational week of delivery was 38 weeks (35–40 weeks). Higher caesarean section rates may be associated with foetal FGR.

The postnatal manifestations of IHPSS vary [24]; moreover, the most common biochemical abnormality in our case was an increase in GGT and bilirubin, which was more common in the multiple-shunt group of live births (80%, 4/5), probably resulting from liver ischaemia due to vascular deprivation. In addition, it should be noted that there were four cases of neonatal hypoglycaemia, all of which were in the single-shunt group. All were prenatally complicated with FGR, including two cases of low birth weight infants and one case of very low birth weight infants. IHPSS can lead to neonatal hypergalactosaemia. Unfortunately, serum galactose was not detected in these four cases; therefore, it was unclear whether hypoglycaemia was caused by transient hypergalactosaemia. There have also been reports of neonatal hypoglycaemia; in a study of the clinical features of congenital portosystemic shunts in the neonatal period, 40% (4/10) of patients experienced severe neonatal hypoglycaemia, and all 4 patients were diagnosed with intrahepatic portosystemic shunts [24]. In our study, hyperammonaemia occurred in 6 neonates, and the blood ammonia level decreased during follow-up observation without causing hepatic encephalopathy. Abdominal ultrasonography in another 2 neonates revealed transient hyperechoic nodules in the liver, which spontaneously subsided during the follow-up process. One of them developed multiple cutaneous haemangiomas, which also subsided spontaneously. Interestingly, both cases were in the multiple-shunt group.

Benign and malignant liver tumours associated with congenital portosystemic shunts have been reported, including focal nodular hyperplasia, hepatocellular adenoma, and hepatoblastoma; however, malignant tumours are only observed in extrahepatic CPSS [25].

Among the cases we followed up, two children underwent laparoscopy and open surgery for shunt ligation, with a good prognosis and no severe postoperative complications. The 10 children with the remaining follow-up adopted conservative treatment, and all of them had spontaneous closure. The average closure time was 8.1 months (1–28 months), which is similar to the conclusion of Achiron [12]. In their cases, the mean interval between delivery and spontaneous closure in the IHPSS was 8.7 months. A management strategy for CPSS proposed that asymptomatic patients with portal–hepatic shunts should be followed up to 2 years old without immediate surgical treatment [2]. Endovascular closure is surgical and the treatment of choice for children who are symptomatic or whose shunt has not closed spontaneously after 2 years of age [26].

Two cases of labour induction (single-shunt group) were found to have copy number variations in the specimens of induction, which were located on chromosomes 7 and X, respectively. The microdeletion on chromosome 7 completely covers the region reported in “Williams-Beuren syndrome” (OMIM 194050). Microduplication located on the short arm of the X chromosome is a variant of unknown clinical significance and does not contain a pathogenic gene included in OMIM. This region is related to the reported region of the “Chromosome Xp11.23-p11.22 duplication syndrome” (OMIM 300,801). Karyotype analysis of the peripheral blood chromosome of one live infant (multiple-shunt group) showed inter-arm inversion of chromosome 9. The inter-arm inversion of chromosome 9 is generally considered a polymorphism; however, some studies have shown that it can have adverse genetic effects. No reports of copy number variations or inter-arm inversions have been found to be associated with IHPSS. Since not all of our cases were examined by whole-genome sequencing, whether these copy number variations or inversions between chromosomal arms were detected incidentally or were associated with the pathogenesis of IHPSS requires further investigation.

Our main limitation was the small number of cases, which will require further large-scale multicentre studies in the future.

## Conclusions

Most IHPSS found during the foetal period is located in the left branch of the portal vein, and the gestational age at diagnosis is usually in the late second or third trimester. The most common concomitant abnormality was FGR. Spontaneous closure of shunts can occur in most live births, and the prognosis is good.

## Data Availability

Anonymised data are available upon request from the authors.

## Abbreviations

ARPV: Anterior right portal vein
CE: Cardiac enlargement
CNVs: Copy number variations
CPSS: Congenital portosystemic shunt
CRL: Crown–rump length
CS: Caesarean section
DI: Duodenal ileus
EIF: Echogenic intracardiac focus
FGR: Foetal growth restriction
GA: Gestational age
GGT: Gamma-glutamyl transferase
HSM: Hepatosplenomegaly
IHPSS: Intrahepatic portosystemic venous shunt
IVC: Inferior vena cava
LB: Live birth
LHV: Left hepatic vein
LPV: Left branch of the portal vein
LPVi: Inferior left portal vein
LPVm: Medial left portal vein
LPVs: Superior left portal vein
MHV: Middle hepatic vein
MS: Multiple shunts
PRPV: Posterior right portal vein
PS: Portal sinus
RHV: Right hepatic vein
RPV: Right branch of the portal vein
SM: Splenomegaly
SS: Single shunt
TOP: Termination of pregnancy
UPSVS: Umbilical–portal–systemic shunt
UV: Umbilical vein
VD: Vaginal delivery
